# Athermal synchronization of laser source with WDM filter in a silicon photonics platform

**DOI:** 10.1063/1.4984022

**Published:** 2017-05-22

**Authors:** Nanxi Li, Zhan Su, Purnawirman, E. Salih Magden, Christopher V. Poulton, Alfonso Ruocco, Neetesh Singh, Matthew J. Byrd, Jonathan D. B. Bradley, Gerald Leake, Michael R. Watts

**Affiliations:** 1 Research Laboratory of Electronics, Massachusetts Institute of Technology, 77 Massachusetts Avenue, Cambridge, Massachusetts 02139, USA; 2 John A. Paulson School of Engineering and Applied Science, Harvard University, 29 Oxford Street, Cambridge, Massachusetts 02138, USA; 3 College of Nanoscale Science and Engineering, University at Albany, 1400 Washington Avenue, Albany, New York 12203, USA

## Abstract

In an optical interconnect circuit, microring resonators (MRRs) are commonly used in wavelength division multiplexing systems. To make the MRR and laser synchronized, the resonance wavelength of the MRR needs to be thermally controlled, and the power consumption becomes significant with a high-channel count. Here, we demonstrate an athermally synchronized rare-earth-doped laser and MRR. The laser comprises a Si_3_N_4_ based cavity covered with erbium-doped Al_2_O_3_ to provide gain. The low thermo-optic coefficient of Al_2_O_3_ and Si_3_N_4_ and the comparable thermal shift of the effective index in the laser and microring cross-sections enable lasing and resonance wavelength synchronization over a wide range of temperatures. The power difference between matched and unmatched channels remains greater than 15 dB from 20 to 50 °C due to a synchronized wavelength shift of 0.02 nm/°C. The athermal synchronization approach reported here is not limited to microring filters but can be applied to any Si_3_N_4_ filter with integrated lasers using rare earth ion doped Al_2_O_3_ as a gain medium to achieve system-level temperature control free operation.

With an increasing demand for data communication bandwidth, integrated silicon photonic technologies have been extensively studied to overcome the limits of electrical interconnects.^1,2^ In an optical interconnect circuit, microring resonators (MRRs) are commonly used as multiplexers/demultiplexers in wavelength division multiplexing (WDM) systems.^3,4^ However, to make the MRR and on-chip laser work together, the resonances of the MRRs should be matched to the laser wavelengths for all operating temperatures.^5,6^ Although the resonance wavelength of a MRR can be controlled by using thermal tuning,^4,7^ the power consumption to align the wavelengths of many MRRs is significant in a high-channel count WDM system.^4,8^ Even when an athermal reference cavity is used, the laser has to be electronically locked to it to prevent any thermally induced mismatch from downstream filters.^9^ To overcome these problems, the MRR filter resonant wavelength can be matched to the laser wavelength and have the same thermal wavelength shift as the laser source.^10,11^ As a result, system-level temperature control-free operation can be achieved without the need for additional tuning power.

Prior to this work, on-chip lasers have been demonstrated using rare-earth elements doped in Al_2_O_3_ as gain media,^12–17^ which has proven to be effective for several reasons. First, rare-earth-doped Al_2_O_3_ glass can be deposited as a single-step back-end-of-line process on silicon wafers without requiring any additional etch steps.^18^ Second, common rare-earth-materials such as Erbium have a wide emission spectrum enabling a wide wavelength tunability and design flexibility.^19–23^ Third, rare-earth-doped gain media do not involve free carriers, unlike semiconductor lasers, and this reduction in losses enables a narrow laser linewidth.^20,22,24^ Furthermore, the low thermo-optic coefficient of the host medium enables operation over a wide temperature range.^25^ Finally, compared to III-V material based systems,^26^ Si_3_N_4_ laser cavities utilizing rare-earth-doped Al_2_O_3_ can have a similar overall thermal response to other Si_3_N_4_ devices on the chip such as MRR filters.^27^ This makes it possible to achieve a control-free operation WDM system consisting of a laser source and a wavelength filter over a wide temperature range.

Here, we demonstrate an Al_2_O_3_:Er^3+^ based distributed feedback (DFB) laser cascaded with Si_3_N_4_ MRRs for filtering different WDM channels on the same chip. The low thermo-optic coefficient of Al_2_O_3_ and Si_3_N_4_^28–31^ and the comparable thermal shift of the effective index in the laser and microring cross-sections enable lasing and resonance wavelength synchronization over a wide range of temperatures. We achieve >15 dB power extinction ratio (between a matched laser-MRR channel and an unmatched channel) from 20 to 50 °C. The wavelength shifts of the laser and the MRRs are synchronized to 0.02 nm/°C, proving that the system is athermal. The athermal synchronization approach reported here is not limited to microring filters but can be applied to any Si_3_N_4_ filter with integrated lasers using rare earth ion doped Al_2_O_3_ as a gain medium to achieve system-level temperature control free operation.

The waveguide cross-section of the DFB laser is shown in Fig. 1(a). The width and gap of the Si_3_N_4_ pieces are selected to be 600 nm and 400 nm, respectively, to provide high mode confinement for both the 1480 nm pump and 1550 nm laser modes within the Al_2_O_3_:Er^3+^ film. The confinement factors in the Al_2_O_3_:Er^3+^ film are calculated to be 84.05% and 84.86% for the fundamental transverse electric (TE) modes at the pump and laser wavelengths, respectively, using a finite-difference 2D mode solver. The height of each Si_3_N_4_ piece is 200 nm. The gap between the Si_3_N_4_ and Al_2_O_3_ layer is 200 nm. A 1100-nm thick Al_2_O_3_:Er^3+^ film is deposited on top to provide gain. The DFB cavity is formed by adding Si_3_N_4_ grating pieces alongside the waveguide with a width of 400 nm, a gap of 500 nm, a duty cycle of 0.5, and a period of 487 nm. The coupling coefficient (κ) is calculated to be 6.4 × 10^2^ m^−1^. The total length of the DFB laser is 2 cm, limited by the maximum length of the chip. For a laser cavity length shorter than 2 cm, with the same pump power, the lasing power decreases. The coupling coefficient and cavity length product (κ·L) is 12.8. At the end of the DFB, a transition is designed to adiabatically couple the mode from the DFB gain waveguide into the mode of a double layer Si_3_N_4_ waveguide. After the transition, the double layer Si_3_N_4_ waveguide is connected to MRRs. The cross-section of the double layer Si_3_N_4_ MRR is shown in the right side of Fig. 1(a). The Si_3_N_4_ pieces in the MRR have a width of 1 *μ*m and a height of 200 nm for each layer, with a 100 nm oxide gap in between. The TE field intensities of the fundamental mode for a DFB gain waveguide and a Si_3_N_4_ MRR are shown in Fig. 1(b), which are calculated from a finite difference mode solver and a bend waveguide mode solver, respectively. The refractive indices used in the mode solver are provided in Fig. 1(c). To ensure mode confinement within the Si_3_N_4_ MRRs, their diameters were selected to be around 90 *μ*m, which corresponds to a free spectral range (FSR) of 5 nm. The entire device is illustrated in Fig. 1(d). Four MRRs after the DFB represent three types of filter channels: one matched, one adjacent, and two low-interfering channels. The diameters of the MRRs are chosen to be 90.02 *μ*m, 90.10 *μ*m, 90.18 *μ*m, and 90.26 *μ*m in order to evenly distribute the resonances within one FSR. The drop ports of the MRRs are used to couple out the laser signal.

**FIG. 1.**
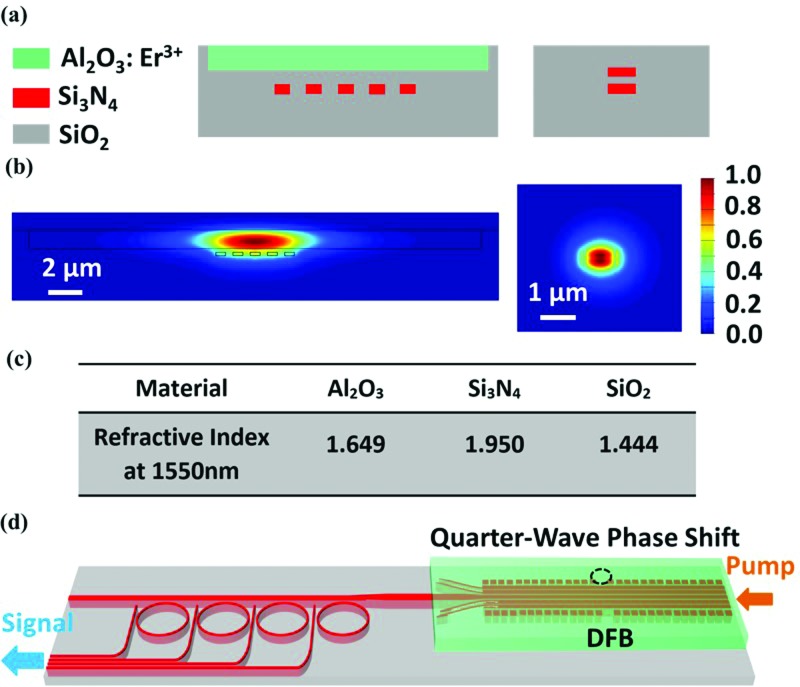
(a) Cross section of the DFB gain waveguide and double layer Si_3_N_4_ MRR. (b) The TE field intensity of the fundamental mode for the DFB gain waveguide and Si_3_N_4_ MRR. (c) Refractive index of the materials at 1550 nm. (d) Sketch of the system, including DFB laser cascaded with Si_3_N_4_ MRRs (not to scale).

The device was fabricated using a 300 mm CMOS foundry. The fabrication process is illustrated in Fig. 2(a). First, a 6-*μ*m thick SiO_2_ bottom cladding layer is deposited on a 300 mm silicon wafer via plasma-enhanced chemical vapor deposition (PECVD). Afterwards, a 200-nm thick PECVD Si_3_N_4_ layer is deposited, followed by a top surface polishing process to reduce the optical scattering loss (Step I). The Si_3_N_4_ layer is subsequently annealed at 1050 °C for 72 min to reduce absorption due to Si-H and N-H bonds around 1.52 *μ*m. Then, the Si_3_N_4_ layer is patterned using 193 nm immersion lithography and reactive ion etching (Step II). The side wall roughness is minimized by a short 900 °C wet oxidation, followed by an HF dip. Next, a 100-nm thick SiO_2_ layer is deposited and the top surface is flattened via chemical-mechanical planarization (CMP), as shown in Step III. Following that step, a second 200-nm-thick Si_3_N_4_ layer is deposited and patterned using the same method as the first layer (Step IV). The fabrication variation in the Si_3_N_4_ pattern will lead to MRR resonance shifts of up to 1 nm, but this has a minimal effect on the DFB lasing wavelength due to the fact that most of the modes is confined in the Al_2_O_3_ film and that the fabrication variations only affect the duty cycle and not the grating period. Above the top Si_3_N_4_ layer, a 4-*μ*m-thick SiO_2_ layer is deposited. The fabrication of the MRRs is completed at Step V, but the DFB laser needs two additional steps. In Step VI, the top SiO_2_ layer is patterned and a 4-*μ*m-deep and 80-*μ*m-wide gain trench is etched using the top Si_3_N_4_ layer as an etch stop. Finally, in Step VII, a 100-nm-thick SiO_2_ layer is deposited within the laser trench and a 1110-nm-thick Al_2_O_3_:Er^3+^ thin film is deposited via reactive co-sputtering. The thickness of the Al_2_O_3_:Er^3+^ film is chosen to ensure that the lasing wavelength matches with one of the Si_3_N_4_ MRR resonances. The deposition of the Al_2_O_3_:Er^3+^ film is a single-step back-end-of-line process on the silicon wafer, allowing direct access to the laser design. Controlling the Al_2_O_3_:Er^3+^ thin film thickness can be used as a general approach to ensure that the lasing wavelength matches with a single channel resonance. Precise control of the DFB lasing wavelength from different wafers has been demonstrated recently in a thulium doped Al_2_O_3_ film.^32^ Deposition runs with different doping levels reveal an optimum Er^3+^ doping concentration of 1.2 × 10^20^ cm^−3^. Given the same pump power, a lower doping concentration will suffer from lower gain while a higher concentration will result in ion clustering or quenching.^33,34^ In order to visualize the Si_3_N_4_ patterning, the top SiO_2_ cladding layer was etched. Scanning electron microscopy (SEM) images of the Si_3_N_4_ patterns (top view) for the DFB laser and a MRR filter are displayed in Figs. 2(b) and 2(c), respectively.

**FIG. 2.**
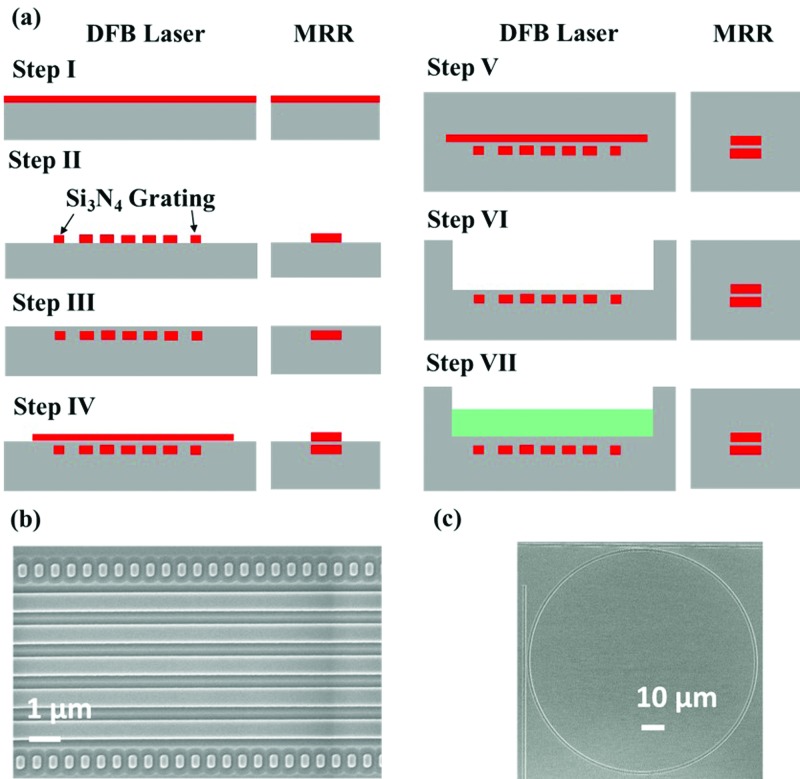
(a) The DFB laser and MRR fabrication steps including (I) deposition and (II) patterning of the bottom Si_3_N_4_ layer, (III) deposition and flattening of the SiO_2_ cladding layer, (IV) deposition and patterning of the top Si_3_N_4_ layer, (V) deposition of the top SiO_2_ cladding layer, (VI) patterning and etching of the top SiO_2_ cladding to form a laser trench, and (VII) deposition of SiO_2_ and Al_2_O_3_:Er^3+^ within the laser trench. (b) and (c) SEM image of the Si_3_N_4_ pattern (top view) for the DFB laser and MRR after removing SiO_2_ top cladding from Step III.

To characterize the system, both external and internal laser measurements were performed on the device. Figure 3(a) shows the setup of the measurement using an external laser, which includes a tunable laser source (Keysight 81600B) to sweep the wavelength of the input signal, a polarization controller to ensure that the input is coupled into the fundamental TE mode of the DFB and MRRs, an optical power meter to record the signals from the drop ports of the MRRs, shown in Figs. 3(b) and 3(c), and a thermoelectric cooler (TEC) to monitor and stabilize the operating temperature of the system with a feedback loop. A cleaved single-mode SMF-28 fiber is used on each side of the chip to butt-couple the tunable laser signal onto the chip and butt-couple the output signal from the chip, respectively.

**FIG. 3.**
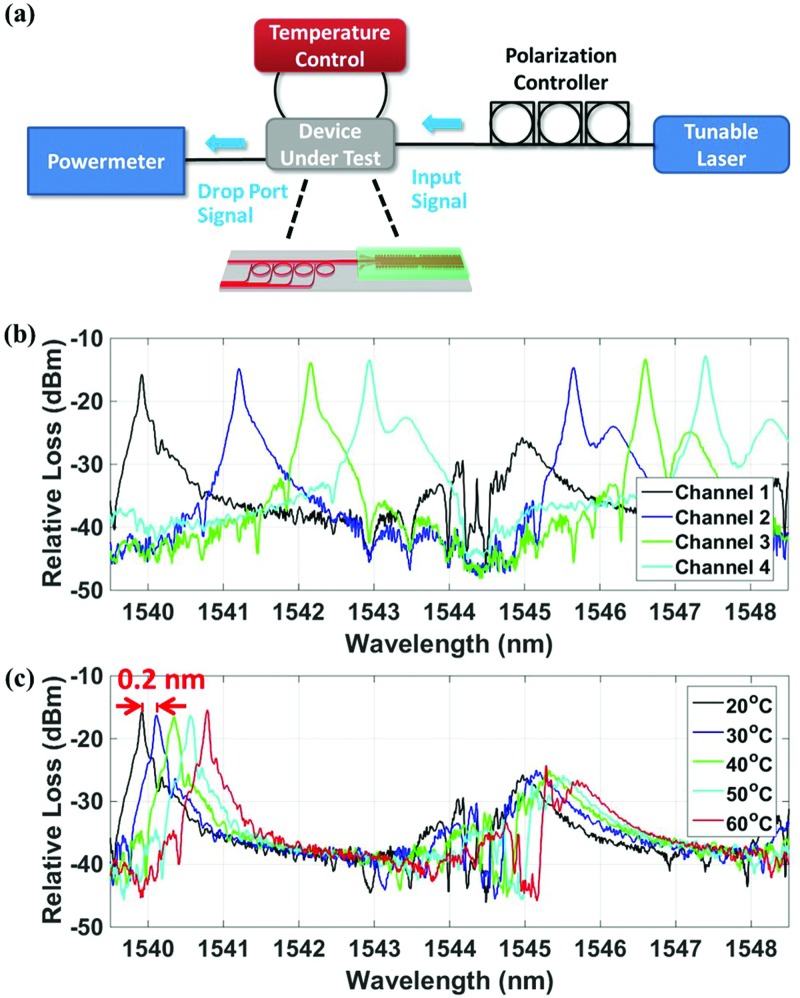
(a) External laser measurement setup including a tunable laser to sweep the wavelength of the input signal, a polarization controller to ensure that the input is coupled into the TE mode of the DFB and MRRs, an optical powermeter to record the signals from the drop port of the MRRs, and a TEC temperature controller to modify, monitor, and stabilize the operating temperature of the system. (b) Passive responses of channels 1, 2, 3, and 4 at 20 °C showing matched, low-interfering, and adjacent channels. (c) The passive response of channel 1 at different temperatures. A 0.2 nm wavelength shift is observed for a 10 °C temperature change from 20 to 50 °C.

Figure 3(b) shows the passive response of each of the four MRR drop ports with an external tunable laser at 20 °C. The main peaks represent the TE resonances of the MRRs, and the smaller lobes on the right side of the peaks are the transverse magnetic (TM) resonance of the MRRs. The loaded quality factor of TE resonance is 2.2 × 10^4^. The existence of the TM mode may be due to the fact that the waveguide after the DFB laser has an asymmetry in the horizontal direction. Under the condition that the transition is not perfectly adiabatic, the TE mode couples into the TM mode. The polarization controller is tuned to couple into the fundamental TE mode of the DFB gain waveguide. For channel 1, only one TE mode peak is observed at 1539.9 nm. At 1544.4 nm, there is no TE mode peak in the system, indicating that the response of the MRR cancels with the DFB cavity response. From the simulation, the MRR corresponding to channel 1 has a resonance at 1544.4 nm, and hence, the DFB lasing signal can have high transmission into this channel. For comparison, the passive response of the DFB cavity together with the MRR drop channels 2, 3, and 4 is shown in Fig. 3(b). For these channels, two orders of TE resonance are observed instead of one. For channels 2 and 3, at the DFB cavity resonance wavelength of 1544.4 nm, the drop ports of the MRRs have low transmission because the channels are not matched. Therefore, these two channels are expected to have less power in the drop port when the on-chip laser with a lasing wavelength of 1544.4 nm is on. While for channel 4, at 1544.4 nm, the transmission is not as low as channels 2 and 3 since it has a tail of the TM mode at this wavelength. Therefore, channel 4 will have a relatively higher output power for the input laser at 1544.4 nm. From Fig. 3(b) and using channel 2, 3, or 4, the FSR of the MRR is measured to be 5 nm. The temperature of the system was then controlled using the TEC, and the passive responses of channel 1 at different temperatures were measured, as shown in Fig. 3(c). A wavelength shift of 0.2 nm is observed for a 10 °C temperature change within a temperature range from 20 to 50 °C. While beyond 50 °C, the wavelength shift becomes larger than 0.2 nm per 10 °C.

The internal on-chip laser measurement setup is shown in Fig. 4. A 1480 nm pump source together with a polarization controller is used to couple the pump signal into the fundamental TE mode of the laser gain waveguide. An external WDM is used to couple out the lasing signal from the pump side of the DFB for analysis. A cleaved SMF-28 fiber is used on each side of the chip to butt-couple the pump onto the chip and output signal from the chip, respectively. Optical spectrum analyzers (OSA) I and II are used to monitor the signal from the DFB laser and MRRs, respectively. A thermoelectric cooler (TEC) is used to modify and monitor the operation temperature of the system. In addition, it also stabilizes the device temperature by reducing the thermal shift due to the pump power.

**FIG. 4.**
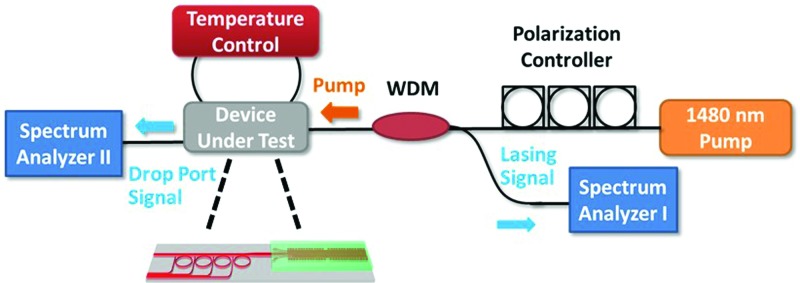
Internal laser measurement setup including a 1480 nm laser pump source together with a polarization controller to ensure that the fundamental TE mode is coupled into the Al_2_O_3_:Er^3+^ DFB laser, optical spectrum analyzers I and II to monitor the DFB laser output and MRR drop port signals, respectively, and a temperature control feedback loop to modify and monitor the temperature of the system.

Figures 5(a) and 5(b) show the output spectra recorded at different temperatures from OSA I and II, respectively. For the DFB laser, a 0.2 nm wavelength shift is observed for a 10 °C temperature change, which matches to the wavelength shift at the output of the MRR shown in Fig. 3(c). Comparing Figs. 5(a) and 5(b), the overall coupling loss from the DFB laser to MRR is 2.5 dB. From Fig. 5(b), the power at the drop port of the matched MRR reduces as the temperature increases, which is caused by the larger wavelength mismatch between the DFB and MRR at higher temperatures of our temperature control range. The wavelength comparison of the DFB and channel 1 MRR is shown in Fig. 5(c). As the temperature increases, the channel 1 MRR has a slightly larger wavelength shift compared to the DFB laser. The relative transmission powers from the four channels are recorded in Fig. 5(d). More than 15 dB power extinction between channel 1 and the rest of the channels is observed within the temperature range from 20 to 50 °C showing athermal operation, which is sufficient for signal processing in a WDM system.^35^ As the temperature is set beyond 50 °C, the power in channel 1 drops by more than 5 dB, due to the wavelength mismatch between the MRR and the DFB, shown in Fig. 5(c). Furthermore, Fig. 5(d) shows that channel 1 has relatively high power and hence it is the matched channel; channels 2 and 3 have relatively low power, and therefore, they are low-interfering channels; channel 4 has medium power so it is the adjacent channel. This matches with the results from the external laser characterization in Fig. 3(b). With the aim of obtaining a more uniform output power over the temperature range from 20 to 50 °C, the DFB laser can be designed to be exactly matched with the resonance of the MRR at 40 °C instead of at 30 °C. Starting at 20 °C, the DFB laser is slightly off the resonance of the MRR. As the temperature increases (to 40 °C, for example), the DFB and MRR resonances will be exactly matched. As the temperature further increases (e.g., 50 °C), the DFB resonance will be slightly off with respect to MRR resonance again. This method is equivalent to shifting the red DFB laser resonance up in Fig. 5(c), so that red and blue points match exactly at 40 °C. In this way, the transmission power will be more uniform over this operation temperature range.

**FIG. 5.**
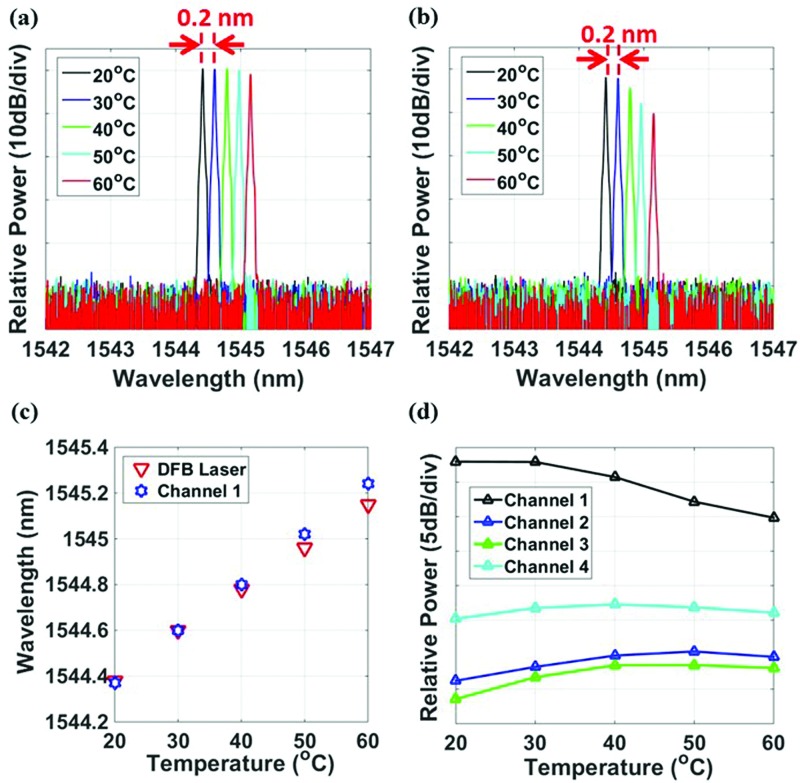
(a) and (b) Optical spectrum of the DFB output (recorded by OSA I) and channel 1 MRR drop port (recorded by OSA II), at different temperatures. (c) Wavelength shift comparison between the DFB laser and channel 1 MRR. (d) Relative power of the different channels over the temperature range of 20 to 50 °C.

In the future, in order to ensure that the resonance of the MRR matches with the laser source on a wafer scale, the design of the MRR filter can be improved. For example, high-order filters or contra-directional couplers can be used to get a flat-top response with wider bandwidth^36,37^ and hence relaxing the requirement of perfect alignment. Additionally, an adiabatic ring design^38^ can be used to make ring more fabrication-tolerant. The work presented in this manuscript acts as a prototype for system-level control-free synchronization while further design effort will offer a wafer-scale production solution. Furthermore, these improved MRRs can be cascaded with multiple grating-based lasers^39^ for a temperature control-free wavelength multiplexer.

In conclusion, we have demonstrated athermally synchronized operation of an integrated DFB laser cascaded with Si_3_N_4_ WDM MRRs on a silicon photonic platform. By making use of the low thermo-optic coefficient of the Al_2_O_3_:Er^3+^ laser gain medium and Si_3_N_4_, athermal operation is demonstrated over a temperature range from 20 to 50 °C. A power extinction ratio of >15 dB between matched and unmatched WDM channels is achieved, and a synchronized wavelength shift of 0.02 nm/°C is reported. The athermal synchronization approach reported here is not limited to microring filters but can be applied to any Si_3_N_4_ filter cascaded with a laser using rare-earth-doped Al_2_O_3_ as a gain medium to achieve system-level temperature control free operation.

## References

[1] D. A. B. Miller , Proc. IEEE 97, 1166–1185 (2009). 10.1109/JPROC.2009.2014298

[2] A. F. Benner , M. Ignatowski , J. A. Kash , D. M. Kuchta , and M. B. Ritter , IBM J. Res. Dev 49, 755–775 (2005). 10.1147/rd.494.0755

[3] P. Rabiei , W. H. Steier , C. Zhang , and L. R. Dalton , J. Lightwave Technol. 20, 1968 (2002). 10.1109/JLT.2002.803058

[4] A. V. Krishnamoorthy , X. Zheng , G. Li , J. Yao , T. Pinguet , A. Mekis , H. Thacker , I. Shubin , Y. Luo , K. Raj , and J. E. Cunningham , IEEE Photonics J. 3, 567–579 (2011). 10.1109/JPHOT.2011.2140367

[5] C. Li , R. Bai , A. Shafik , E. Z. Tabasy , B. Wang , G. Tang , C. Ma , C. H. Chen , Z. Peng , M. Fiorentino , R. G. Beausoleil , P. Chiang , and S. Palermo , IEEE J. Solid-State Circuits 49, 1419–1436 (2014). 10.1109/JSSC.2014.2321574

[6] S. Tanaka , T. Akiyama , S. Sekiguchi , and K. Morito , Fujitsu Sci. Tech. J. 50, 123–131 (2014), available at https://www.fujitsu.com/global/documents/about/resources/publications/fstj/archives/vol50-1/paper19.pdf.

[7] C. Sun , M. T. Wade , Y. Lee , J. S. Orcutt , L. Alloatti , M. S. Georgas , A. S. Waterman , J. M. Shainline , R. R. Avizienis , S. Lin , B. R. Moss , R. Kumar , F. Pavanello , A. H. Atabaki , H. M. Cook , A. J. Ou , J. C. Leu , Y.-H. Chen , K. Asanović , R. J. Ram , M. A. Popović , and V. M. Stojanović , Nature 528, 534–538 (2015). 10.1038/nature16454 26701054

[8] M. S. Dahlem , C. W. Holzwarth , A. Khilo , F. X. Kärtner , H. I. Smith , and E. P. Ippen , Opt. Express 19, 306–316 (2011). 10.1364/OE.19.000306 21263570

[9] E. S. Magden , M. Y. Peng , J. D. B. Bradley , G. Leake , D. Coolbaugh , L. A. Kolodziejski , F. X. Kaertner , and M. Watts , “ Laser frequency stabilization using Pound-Drever-Hall technique with an integrated TiO_2_ athermal resonator,” in *Conference on Lasers and Electro-Optics (CLEO) 2016*, San Jose, California (2016), p. STu1H.3 10.1364/CLEO_SI.2016.STu1H.3

[10] J. Seok-Hwan , T. Shinsuke , S. Shigeaki , K. Teruo , H. Nobuaki , A. Suguru , U. Tatsuya , Y. Tsuyoshi , A. Tomoyuki , T. Yu , and M. Ken , Jpn. J. Appl. Phys., Part 1 51, 082101 (2012). 10.1143/JJAP.51.082101

[11] S. Tanaka , S.-H. Jeong , S. Sekiguchi , T. Kurahashi , Y. Tanaka , and K. Morito , Opt. Express 20, 28057–28069 (2012). 10.1364/OE.20.028057 23263042

[12] G. Singh , Purnawirman , J. D. B. Bradley , N. Li , E. S. Magden , M. Moresco , T. N. Adam , G. Leake , D. Coolbaugh , and M. R. Watts , Opt. Lett. 41, 1189–1192 (2016). 10.1364/OL.41.001189 26977666

[13] Z. Su , N. Li , E. Salih Magden , M. Byrd , Purnawirman , T. N. Adam , G. Leake , D. Coolbaugh , J. D. B. Bradley , and M. R. Watts , Opt. Lett. 41, 5708–5711 (2016). 10.1364/OL.41.005708 27973495

[14] M. Belt and D. J. Blumenthal , Opt. Express 22, 10655–10660 (2014). 10.1364/OE.22.010655 24921766

[15] J. D. B. Bradley , Z. Su , E. S. Magden , N. Li , M. Byrd , Purnawirman , T. N. Adam , G. Leake , D. Coolbaugh , and M. R. Watts , “ 1.8-*μ*m thulium microlasers integrated on silicon,” Proc. SPIE 9744, 97440U (2016). 10.1117/12.2213678 27973495

[16] Z. Su , J. D. B. Bradley , N. Li , E. S. Magden , P. Purnawirman , D. Coleman , N. Fahrenkopf , C. Baiocco , T. N. Adam , G. Leake , D. Coolbaugh , D. Vermeulen , and M. R. Watts , “ Ultra-compact CMOS-compatible ytterbium microlaser,” in *Advanced Photonics 2016 (IPR, NOMA, Sensors, Networks, SPPCom, SOF)*, Vancouver (2016), p. IW1A.3 10.1364/IPRSN.2016.IW1A.3

[17] M. Belt , T. Huffman , M. L. Davenport , W. Li , J. S. Barton , and D. J. Blumenthal , Opt. Lett 38, 4825–4828 (2013). 10.1364/OL.38.004825 24322142

[18] E. S. Magden , Purnawirman , N. Li , G. Singh , J. D. B. Bradley , G. S. Petrich , G. Leake , D. D. Coolbaugh , M. R. Watts , and L. A. Kolodziejski , “ Fully CMOS-compatible integrated distributed feedback laser with 250 °C fabricated Al_2_O_3_:Er^3+^ Gain Medium,” in *Conference on Lasers and Electro-Optics (CLEO)* (2016), p. SM1G.2 10.1364/CLEO_SI.2016.SM1G.2

[19] S. Li , D. Zhang , J. Zhao , Q. Yang , X. Xiao , S. Hu , L. Wang , M. Li , X. Tang , Y. Qiu , M. Luo , and S. Yu , Opt. Express 24, 6341–6349 (2016). 10.1364/OE.24.006341 27136825

[20] N. Li , E. Timurdogan , C. V. Poulton , M. Byrd , E. S. Magden , Z. Su , Purnawirman , G. Leake , D. D. Coolbaugh , D. Vermeulen , and M. R. Watts , Opt. Express 24, 22741–22748 (2016). 10.1364/OE.24.022741 27828950

[21] Y. Liu , K. Wu , N. Li , L. Lan , S. Yoo , X. Wu , P. P. Shum , S. Zeng , and X. Tan , J. Opt. Soc. Korea 17, 357–361 (2013). 10.3807/JOSK.2013.17.5.357

[22] Y. W. Song , S. A. Havstad , D. Starodubov , Y. Xie , A. E. Willner , and J. Feinberg , IEEE Photonics Technol. Lett. 13, 1167–1169 (2001). 10.1109/68.959352

[23] J. H. Wong , H. Q. Lam , S. Aditya , J. Zhou , N. Li , J. Xue , P. H. Lim , K. E. K. Lee , K. Wu , and P. P. Shum , J. Lightwave Technol. 30, 3164–3172 (2012). 10.1109/JLT.2012.2215008

[24] E. H. Bernhardi , H. A. G. M. van Wolferen , L. Agazzi , M. R. H. Khan , C. G. H. Roeloffzen , K. Wörhoff , M. Pollnau , and R. M. de Ridder , Opt. Lett 35, 2394–2396 (2010). 10.1364/OL.35.002394 20634841

[25] M. Belt and D. J. Blumenthal , “ High temperature operation of an integrated erbium-doped DBR laser on an ultra-low-loss Si3N4 platform,” in *Optical Fiber Communications Conference and Exhibition (OFC)* (2015), pp. 1–3. 10.1364/OFC.2015.Tu2C.7

[26] A. W. Fang , E. Lively , Y.-H. Kuo , D. Liang , and J. E. Bowers , Opt. Express 16, 4413–4419 (2008). 10.1364/OE.16.004413 18542537

[27] Purnawirman , E. S. Hosseini , M. Moresco , Z. Su , E. Timurdogan , A. Baldycheva , J. Sun , M. R. Watts , T. N. Adam , G. Leake , and D. Coolbaugh , “ Integrated Al_2_O_3_:Er^3+^ DFB laser for temperature control free operation with silicon nitride ring filter,” in *Conference on Lasers and Electro-Optics (CLEO)*, San Jose, California (2014), p. SM4G.5 10.1364/CLEO_SI.2014.SM4G.5

[28] S. Wiechmann and J. Müller , Thin Solid Films 517, 6847–6849 (2009). 10.1016/j.tsf.2009.05.040

[29] M. R. Saleem , R. Ali , S. Honkanen , and J. Turunen , Thin Solid Films 542, 257–262 (2013). 10.1016/j.tsf.2013.06.030

[30] A. Arbabi and L. L. Goddard , Opt. Lett. 38, 3878–3881 (2013). 10.1364/OL.38.003878 24081076

[31] A. W. Elshaari , I. E. Zadeh , K. D. Jöns , and V. Zwiller , IEEE Photonics J. 8, 1–9 (2016). 10.1109/JPHOT.2016.2561622

[32] N. Li , P. Purnawirman , Z. Su , E. Salih Magden , P. T. Callahan , K. Shtyrkova , M. Xin , A. Ruocco , C. Baiocco , E. P. Ippen , F. X. Kärtner , J. D. B. Bradley , D. Vermeulen , and M. R. Watts , Opt. Lett. 42, 1181–1184 (2017). 10.1364/OL.42.001181 28295078

[33] L. Agazzi , K. Wörhoff , and M. Pollnau , J. Phys. Chem. C 117, 6759–6776 (2013). 10.1021/jp4011839

[34] N. Li , Purnawirman , J. D. B. Bradley , G. Singh , E. S. Magden , J. Sun , and M. R. Watts , “ Self-pulsing in Erbium-doped fiber laser,” in *Optoelectronics Global Conference (OGC)* (2015), pp. 1–2. 10.1109/OGC.2015.7336833

[35] F. Horst , W. M. J. Green , S. Assefa , S. M. Shank , Y. A. Vlasov , and B. J. Offrein , Opt. Express 21, 11652–11658 (2013). 10.1364/OE.21.011652 23736388

[36] P. Chen , S. Chen , X. Guan , Y. Shi , and D. Dai , Opt. Lett. 39, 6304–6307 (2014). 10.1364/OL.39.006304 25361340

[37] W. Shi , X. Wang , C. Lin , H. Yun , Y. Liu , T. Baehr-Jones , M. Hochberg , N. A. F. Jaeger , and L. Chrostowski , Opt. Express 21, 3633–3650 (2013). 10.1364/OE.21.003633 23481820

[38] J. C. Mikkelsen , W. D. Sacher , and J. K. S. Poon , Opt. Express 22, 9659–9666 (2014). 10.1364/OE.22.009659 24787851

[39] Purnawirman , N. Li , E. S. Magden , G. Singh , M. Moresco , T. N. Adam , G. Leake , D. Coolbaugh , J. D. B. Bradley , and M. R. Watts , Opt. Lett. 42, 1772–1775 (2017). 10.1364/OL.42.001772 28454157

